# Developmental cost of leg-regenerated *Coccinella septempunctata* (Coleoptera: Coccinellidae)

**DOI:** 10.1371/journal.pone.0210615

**Published:** 2019-01-18

**Authors:** Pengxiang Wu, Fengming Wu, Shuo Yan, Chang Liu, Zhongjian Shen, Xiaofei Xiong, Zhen Li, Qingwen Zhang, Xiaoxia Liu

**Affiliations:** 1 Department of Entomology, China Agricultural University, Beijing, China; 2 Entomology and Nematology Department, University of Florida, Gainesville, FL, United States of America; Nigde Omer Halisdemir University, TURKEY

## Abstract

As larval cannibalism is common under intensive rearing conditions, leg regeneration can help ladybugs adapt to the competitive environment, but whether the leg regeneration leads to side effects on development remains unclear. To analyze the potentially developmental cost of leg regeneration, the developmental period and weight of leg-regenerated *Coccinella septempunctata* were studied in the laboratory. The results showed that, when the time intervals between the emergency of 4th-instar larva and leg amputation increased, the developmental period of leg-regenerated 4th-instar larvae was gradually prolonged. Significantly developmental delay were also examined at prepupal and pupal stages, and various timings of leg amputation affected the periods of leg-regenerated prepupae/pupae similarly. After the leg was amputated at different larval instars, the developmental delay only occurred at the larval instar when the leg was amputated, whereas other larval instars failed to be extended, and the developmental periods of leg-regenerated prepupae/pupae were affected similarly by the instars of leg amputation. Developmental delays possibly resulted in more consumption by leg-regenerated larvae, and then weight gains at prepupal/pupal stages, but different larval instars of leg amputation affected the weight gain similarly. Both the developmental delay (at 4th-instar larval, prepupal and pupal stages) and weight gain (at pupal and adult stages) in complete/bilateral amputation were longer or greater than those in half/unilateral amputation. However, the thoracic locations of leg amputation impacted the developmental delay and weight gain similarly. Our study indicates that although leg regeneration triggers the developmental cost decreasing the competitive superiority or agility, *C*. *septempunctata* larvae still choose to completely regenerate the leg to adapt to complex environments. Thus, in order to remain competitive at adult stages, leg-impaired larvae may make an investment tradeoff between leg regeneration and developmental cost.

## Introduction

Regeneration is a process of regrowing or renovating injured tissues/cells in organisms [1,2], and the in-depth study of regeneration may contribute to technical improvements in repairing damaged human organs [3]. Epimorphosis observed in both vertebrate and invertebrate is the tissue reestablishment of lost parts via cell multiplication [4,5]. Rebuilding impaired appendages is common across many taxonomic groups such as malacostracan crustaceans [6] and insects [7,8], contributing to researches in various respects [9] containing genetic, cellular, tissue, organic and organismal mechanisms. Leg regeneration in insects has been studied in at least 36 genera of 11 orders, including Blattaria, *Periplaneta americana* [10], *Leucophaea maderae* [11], *Eupolyphaga sinensis* Walker [12], Phasmida, *Sinophasma* spp. [13], Orthoptera, *Acheta domestica* [14], Lepidoptera, *Galleria mellonella* [15], Odonata, *Ischnura cervula* [16], Dictyoptera, *Blattella germanica* [17], Triatominae, *Rhodnius prolixus* [18], Heteroptera, *Oncopeltus fasciatus* [19], Coleoptera [20,21,22], *Tribolium castaneum* [23]. Numerous studies associated with regeneration are also reported in hemimetabolous insects such as cockroaches [24,25] and crickets [26,27]. Leg regeneration helps insects adapt to competitive environments, but also causes developmental costs.

Different forms of organ/tissue damage cause a systemic reaction of insects, and thus extend developmental periods. Mini-incisions to the integument of Galleria extend the larval duration by nearly a day [28], and leg amputations also lead to developmental delays of Blattella and Periplaneta [29,30,31,32]. The regeneration of injured legs influences the molt cycle of insects, resulting in the molting delay [33,34,35,36,37,38,39,40,41,42,43]. Many insects have a capability to heal impaired legs through localized cell proliferation [44]. Insulin-like peptides secreted by impaired imaginal tissues of Drosophila act as a signal inhibiting ecdysteroid production [45,46,47,48,49,50,51], causing developmental delays [52,53,54]. The development may be suspended until the injured cells/tissues are regrown and then intact morphologies appear [55,56]. Moreover, the pupal stage is also affected by the damage, larval Drosophila injured in 48–84 hours delays the pupariation [57,58]. Ecdysteroids can control the period of developmental transitions including larval-larval, larval-pupal and pupal-adult molts [59,60,61].

The degree of developmental delay depends on two potential factors: the amount of impaired tissue/cell and the developmental stage when damage occurs [62,63,64,65,66]. Besides delays at larval stages, the pupariation delay is also positively correlated with the amount of injured larval tissues/cells [67,68]. Similarly, after fragments of wing imaginal discs are implanted into Ephestia larvae, the fragments regenerate completely, leading to pupariation delays [41]. Moreover, injures at different developmental timings have different effects [56,60,61,62]. For example, imaginal disc fragments are transplanted into Ephestia at various larval stages, triggering different degrees of developmental delay [42]. Developmental delays make *Harmonia axyridis* invest more nutrient resources into regeneration, and increased consumption results in weight gains [21].

Under intensive rearing conditions, losing a leg is common for coccinellid larvae due to cannibalism. Although lost legs caused by cannibalism could be regenerated, the development of ladybugs may be affected. Thus in the current study, the developmental cost of leg-regenerated *Coccinella septempunctata* was examined. Our goals in this study were to determine: 1) whether the leg amputation at different timings of 4th-instar caused the developmental delay of leg-regenerated ladybugs. 2) Whether the factors containing the instar, site, thoracic location and amount of leg amputation influenced the developmental period and weight of leg-regenerated ladybugs.

## Materials and methods

### Insects

Ladybugs *C*. *septempunctata* were taken from our laboratory colony (Laboratory of Integrated Pest Management, China Agricultural University, Beijing, China). Larvae were kept in plastic containers (7 cm × 4.5 cm × 8 cm), reared with fresh bean aphids (*Acyrthosiphon pisivorum*) under the condition of a constant temperature of 25 ± 1°C, RH = 60–70% and a photoperiod of 16: 8 (L: D) under the light intensity of 600 lux.

### Effect of leg amputation at different timings of 4th-instar on the developmental period of leg-regenerated ladybugs

To evaluate whether timings of leg amputation impacted development periods of leg-regenerated ladybugs, larvae at the 4th-instar rather than other instars were selected due to the longer duration. Zero-, 0.5-, 1-, 1.5-, 2-, 2.5-, 3-, 3.5- or 4 days after the 4th-instar larva emerged, its left-middle leg was amputated at the base of the tibia (half amputation), so each group contained 9 treatments. After anesthetization, the larva was placed on a double-sided tape, and the leg was amputated using a pair of micro-scissors. Then the leg-amputated larvae were held and fed in the same conditions as mentioned above. The 4th-instar larval, prepupal and pupal periods were recorded. Emerging adults were tested by microscopy to determine whether the amputated leg was regenerated again (the lost part reappeared). Moreover, ladybugs without any treatments were regarded as control. Each treatment was replicated three times simultaneously, and each replication included 20 ladybugs (male: female = 1: 1).

### Effects of instar, site, thoracic location and amount of leg amputation on the developmental period and weight of leg-regenerated ladybugs

**Fig 1:**
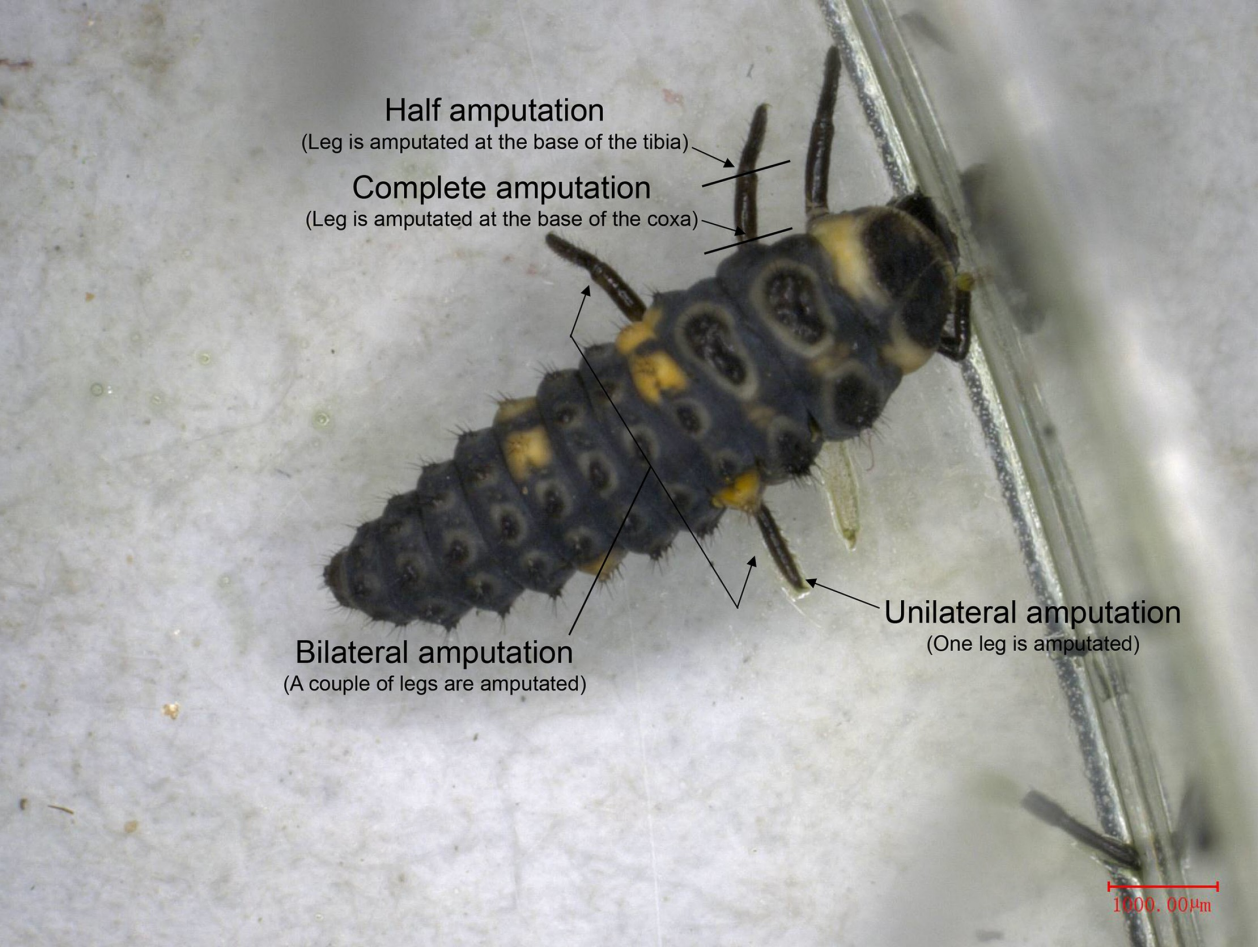
Complete amputation: The larval leg of *C*. *septempunctata* was amputated at the base of the coxa. Half amputation: the larval leg was amputated at the base of the tibia. Unilateral amputation: one larval leg was amputated. Bilateral amputation: a pair of legs was amputation. Scale bars equal 1000 μm.

To analyze the factors influencing the developmental period and weight of leg-regenerated ladybugs, 1) the left-middle leg of larvae in three instars (2nd-, 3rd- or 4th-instar) was amputated at the base of the tibia (since 1st-instar larvae exhibited high mortality rates after leg amputation); 2) the left-middle leg of 4th-instar larvae was amputated at the base of the tibia (half amputation) or coxa (complete amputation); 3) the leg of 4th-instar larvae was half amputated at 3 thoracic locations (fore-, mid-, or hind leg); 4) the left-middle leg of 4th-instar larvae was half amputated in different amounts (unilateral or bilateral amputation) (Fig 1). Developmental periods of leg-regenerated larvae, prepupae and pupae were recorded, and the leg-regenerated pupae and adults were weighted using analytical balance (BS124S, Sartorius, Goettingen, Germany). Ladybugs without any treatments were regarded as control. Each treatment contained 20 ladybugs (male: female = 1: 1) and was replicated for three times simultaneously.

### Statistical analysis

Descriptive statistics were given as the mean values and standard errors of the mean. Differences in developmental periods or weights between two treatments were examined using independent t-tests. Other data were analyzed using one-way ANOVA with the post hoc Tukey’s honest test of significance at the 5% level of statistical significance. All statistical analyses were conducted using the SPSS 20.0 software (IBM, Armonk, NY).

## Results

### Effect of leg amputation at different timings of 4th-instar on the developmental period of leg-regenerated ladybugs

**Fig 2:**
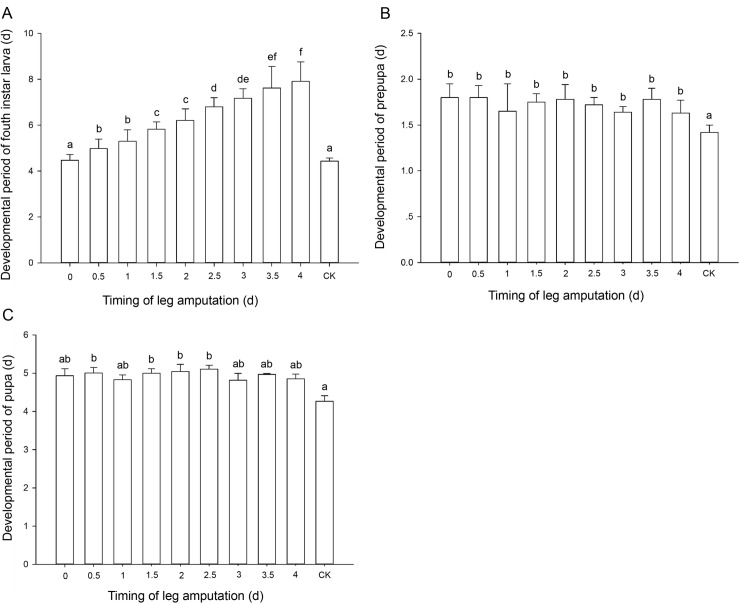
Mean (± SE) developmental periods (d) of leg-regenerated ladybugs at various stages after the leg was amputated at different timings of 4th-instar. A. Fourth instar larva; B. Prepupa; C. Pupa. Different letters indicate significant differences among the treatments (Tukey’s HSD, *P* < 0.05).

Developmental periods of leg-regenerated 4th-instar larvae were prolonged progressively when the leg was amputated from day 0 to day 4, and almost all 4th-instar larval periods of leg-regenerated ladybugs significantly longer than that of control (*F*_9, 38_ = 155.556, *P* < 0.001; Fig 2A). Various timings of leg amputation affected developmental periods of leg-regenerated prepupae/pupae similarly, but both leg-regenerated prepupae (*F*_9, 20_ = 2.895, *P* = 0.014; Fig 2B) and pupae (*F*_9, 20_ = 2.831, *P* = 0.025; Fig 2C) delayed development significantly compared to normal individuals after the leg was amputated at the different timings.

### Effects of instar, site, thoracic location and amount of leg amputation on the developmental period and weight of leg-regenerated ladybugs

#### Developmental period

**Fig 3:**
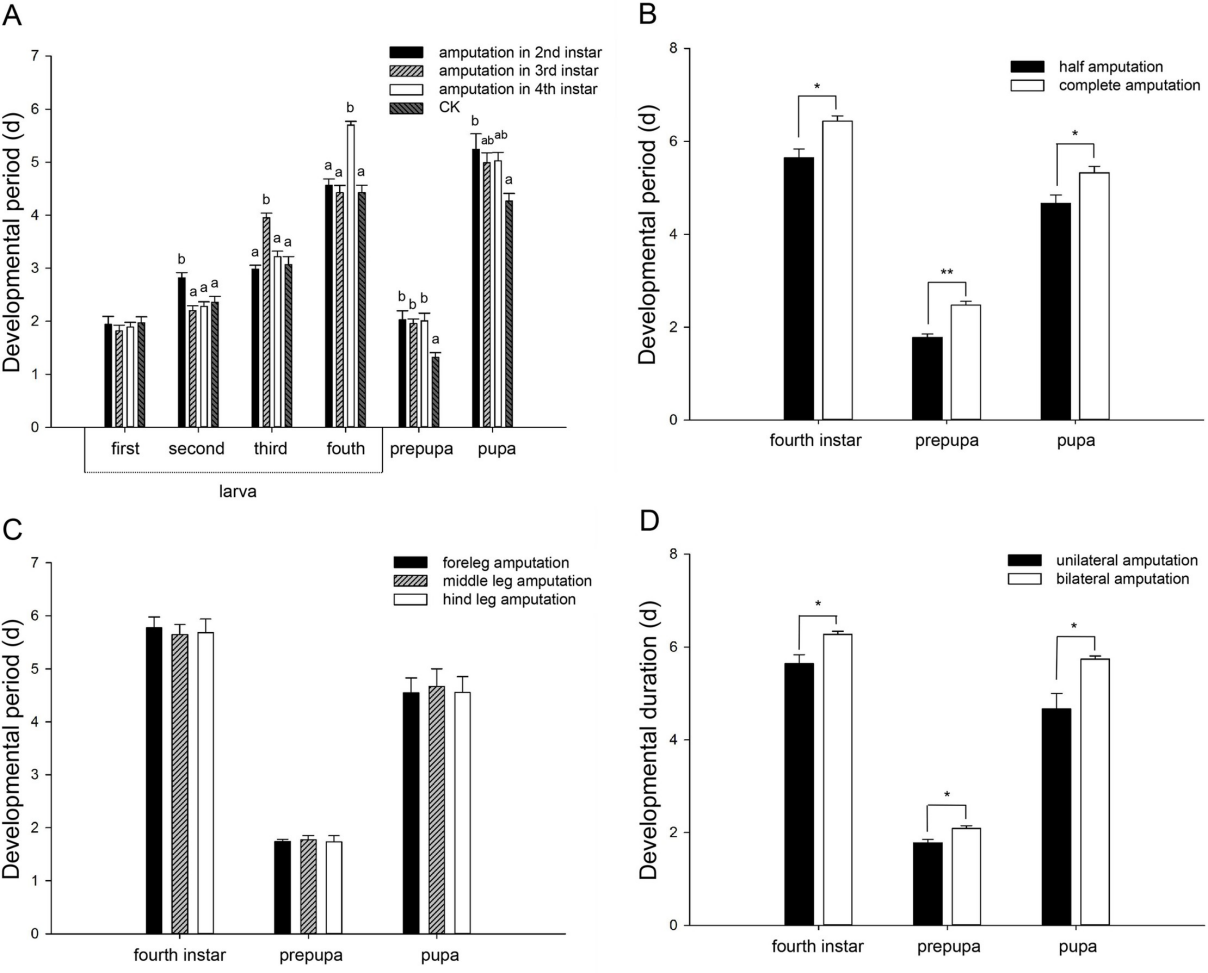
Mean (± SE) developmental periods (d) of leg-regenerated ladybugs at 4th-instar larval, prepupal and pupal stages. A. Results in amputation at various larval stages, i.e., the 2nd-, 3rd- or 4th instar, together with CK (normal developmental duration); B. Results in half and complete amputations; C. Results in amputations at various thoracic locations (fore-, mid- or hind leg); D. Results in unilateral and bilateral amputations. Different letters indicate significant differences among the treatments (Tukey’s HSD, *P* < 0.05). Asterisks indicate significant differences between two treatments (independent t-test, *P < 0.05; **P < 0.01).

After the leg was amputated at various larval instars, only the larval instar when the leg was amputated was significantly extended. Compared to normal periods, developmental periods of leg-regenerated 2nd-, 3rd- and 4th-instar larvae were prolonged significantly when the leg was amputated at the 2nd- (*F*_3, 8_ = 8.373, *P* = 0.008), 3rd- (*F*_3, 8_ = 16.265, *P* = 0.001) and 4th- (*F*_3, 8_ = 26.821, *P* < 0.001) instars, respectively. Moreover, the developmental periods of leg-regenerated prepupa and pupa were impacted similarly by the instars of leg amputation, and both of them were extended significantly compared to normal periods (prepupa, *F*_3, 8_ = 7.357, *P* = 0.011; pupa, *F*_3, 8_ = 4.279, *P* = 0.044; Fig 3A). Developmental delays of leg-regenerated 4th-instar larvae (*t*_4_ = 3.551, *P* = 0.024), prepupae (*t*_4_ = 6.283, *P* = 0.003) or pupae (*t*_4_ = 2.895, *P* = 0.044) in complete amputation were significantly longer than those in half amputation (Fig 3B). However, the developmental periods at 4th-instar larval (*F*_2, 6_ = 0.102, *P* = 0.904), prepupal (*F*_2, 6_ = 0.082, *P* = 0.922) and pupal (*F*_2, 6_ = 0.048, *P* = 0.954) stages were affected similarly by thoracic locations of leg amputation, i.e., the fore-, mid- or hind leg (Fig 3C). Both bilateral and unilateral amputations caused developmental delays of leg-regenerated ladybugs, and the 4th-instar larval (*t*_4_ = 3.077, *P* = 0.037), prepupal (*t*_4_ = 3.202, *P* = 0.033) and pupal (*t*_4_ = 3.141, *P* = 0.035) periods in bilateral amputation were significantly longer than those in unilateral amputation (Fig 3D).

#### Weight

**Fig 4:**
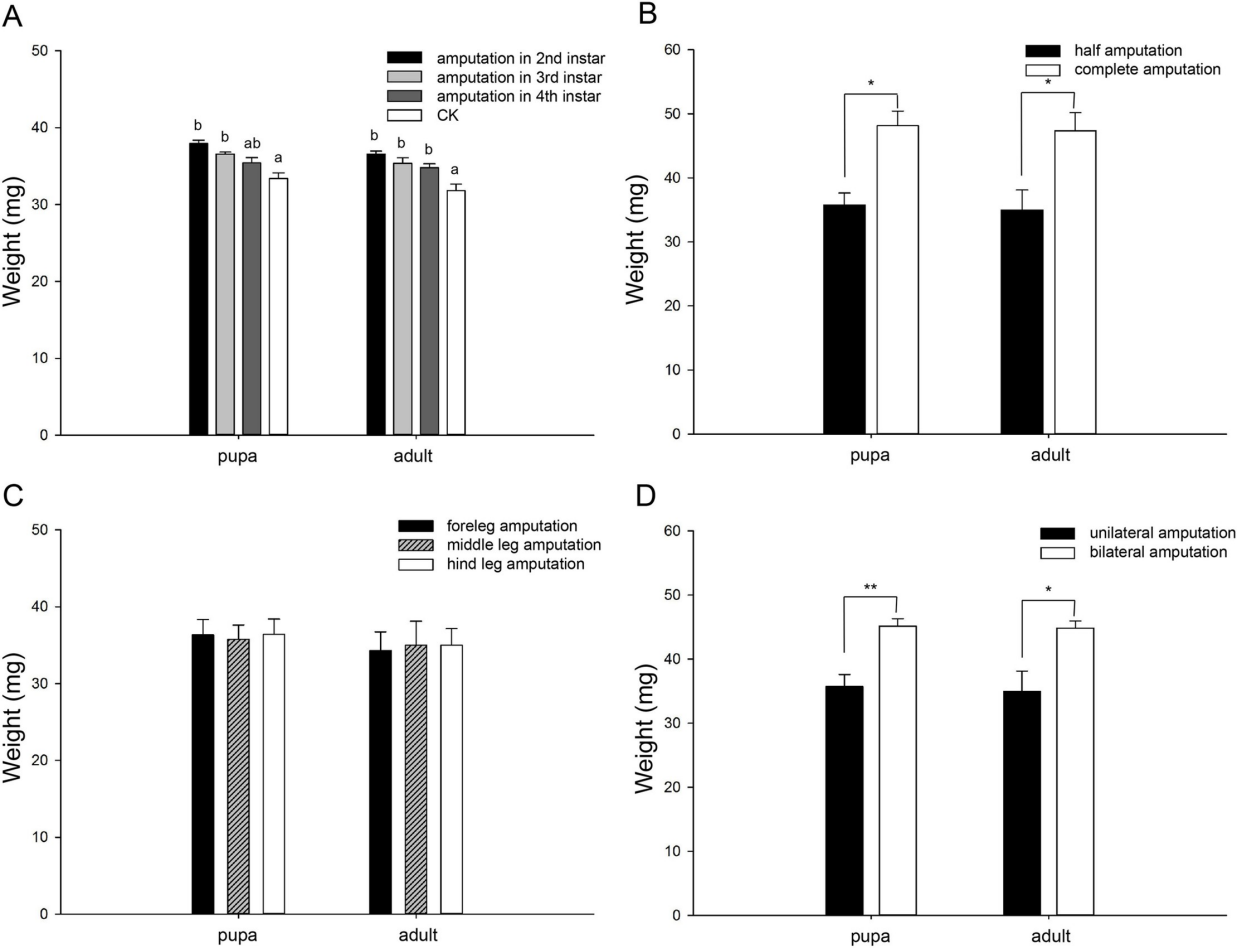
Mean (± SE) weights (mg) of leg-regenerated ladybugs at pupal and adult stages. A. Results in amputations at the 2nd-, 3rd- or 4th instar larval stage, together with CK (normal weight); B. Results in half and complete amputations; C. Results in amputations at various thoracic locations (fore-, mid- or hind leg); D. Results in unilateral and bilateral amputations. Different letters indicate significant differences among the treatments (Tukey’s HSD, *P* < 0.05). Asterisks indicate significant differences between two treatments (independent t-test, *P < 0.05; **P < 0.01).

When the leg of ladybugs was amputated at various larval instars, the weights of leg-regenerated pupae/adults were impacted similarly by the instars of leg amputation, and both the leg-regenerated pupae (*F*_3, 8_ = 11.696, *P* = 0.003) and adults (*F*_3, 8_ = 9.774, *P* = 0.005) gained weights significantly compared to normal individuals (Fig 4A). Weight gains of leg-regenerated pupae (*t*_4_ = 4.26, *P* = 0.013) or adults (*t*_4_ = 2.939, *P* = 0.042) in complete amputation were significantly greater than those in half amputation (Fig 4B). Nevertheless, pupal (*F*_2, 6_ = 0.033, *P* = 0.967) or adult (*F*_2, 6_ = 0.025, *P* = 0.975) weights were affected similarly among various thoracic locations of leg amputation (Fig 4C). Leg-regenerated pupae (*t*_4_ = 4.732, *P* = 0.009) or adults (*t*_4_ = 2.808, *P* = 0.048) gained greater weights in bilateral amputation compared to those in unilateral amputation (Fig 4D) (All data from figures are provided in S1 Table).

## Discussion

### Developmental delay of leg-regenerated ladybugs

Some species such as crab and scolopendra shorten developmental periods during leg regeneration [12,69], whereas many species including Drosophila, Ephestia and Galleria delay development after the leg was impaired [15,31,45,55,61,70]. Since larval cannibalism is frequent in intensive rearing systems, regenerating lost legs is common for *C*. *septempunctata* to adapt to the competitive environment, but in the meantime the normal development of this beneficial species is impacted. Developmental delays of leg-regenerated ladybugs were observed when the leg was amputated at various timings of 4th-instar, and developmental periods of leg-regenerated 4th-instar larvae were gradually prolonged with increased intervals between the emergence of 4th-instar larva and leg amputation, suggesting that development might be suspended until the damaged leg was regrown [55,56], so the timing of leg amputation impacted the degree of developmental delay [60,61]. It is also found that in *Blattella germanica* and *Periplaneta americana*, the leg amputation delayed the subsequent molt until wound healing and complete regeneration of the leg [30,68]. Insulin-like peptides secreted by damaged tissues act as a signal inhibiting ecdysteroid production, impacting normal larval-larval molts [51,52]. However, developmental delays were also tested at subsequently prepupal and pupal stages, implying that ecdysteroid controlled not only larval-larval molts but also larval-pupal molts [60,61].

### Developmental period and weight of leg regenerated ladybugs are affected by the instar, site and amount of leg amputation

When the leg was amputated at different larval instars, the obvious prolongation was only detected at the larval instar when leg amputation occurred, whereas other larval instars were not affected, indicating that the ecdysteroid might independently control molt cycle in each larval instar, causing that the developmental delay in each larval instar was independently impacted by leg amputation [71,72]. Furthermore, the degree of developmental delay at subsequently prepupal and pupal stages was affected similarly by larval instars of leg amputation. The consumption ratio of leg-regenerated/normal larvae was 1.103 (Wu unpublished data), so development delays might increase consumption by leg-regenerated larvae. Thus at a later stage, the leg-regenerated pupae and adults also significantly gained weights after the leg was amputated at different larval stages, further indicating extended time might be spent accumulating resources for leg regeneration and growth [9]. But on the other hand, weight gains may decrease the agility of ladybugs, damaging the competitive ability. Weight gains can be examined not only in insects, but also in other arthropods such as cellar spiders *Holocnemus pluchei* [73] and American lobster *Homarus americanus* [74].

The degree of developmental delays depends on not only the developmental stage of leg amputation, but also the amount of impaired tissues or cells [62,63,64]. In Periplaneta and Blattella, the amputation of a second leg causes an extra delay after a single leg is amputated [29,30,31,32,68]. Compared to half or unilateral amputation, more tissues are damaged in complete or bilateral amputation, so more time is spent on leg regeneration. Thus, developmental delays of leg-regenerated 4th-instar larvae, prepupae and pupae in complete/bilateral amputation were longer than those in half/unilateral amputation. Moreover, the degree of the developmental delays was impacted similarly among thoracic locations of leg amputation. Besides leg-amputation treatment, X-irradiation, ethyl methanesulfonate treatment and electromagnetic field also support the notion that the extent of delay is positively correlated with the amount of injured cells or tissues [46,60,61,65]. More damaged cells or tissues lead to longer developmental delays, causing more consumption. Thus, similar to developmental delays, weight gains of leg-regenerated pupae and adults in complete/bilateral amputation were greater than those in half/unilateral amputation.

### Asymmetry of leg regeneration after bilateral amputation

**Fig 5:**
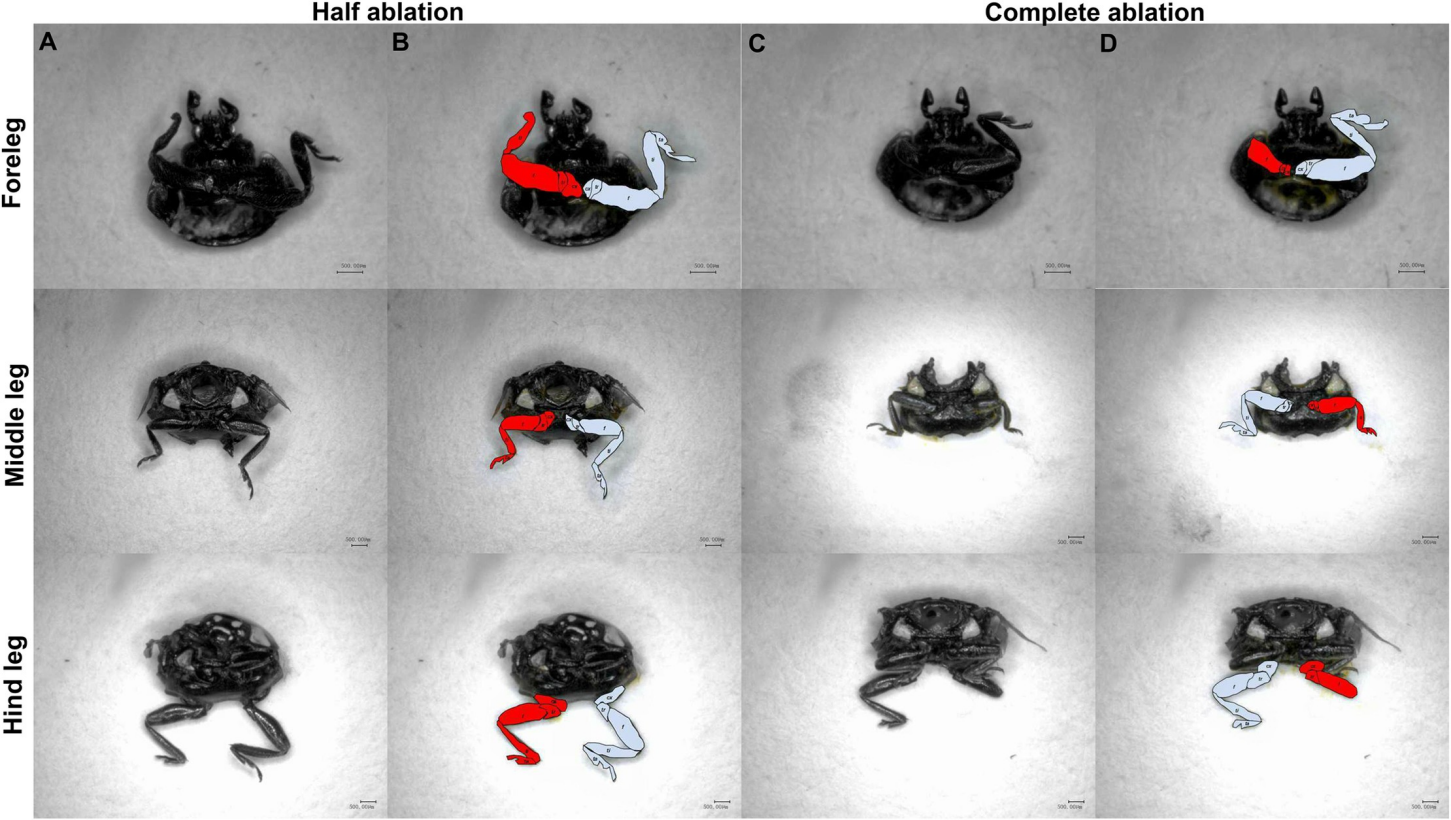
Regeneration of fore-, mid- or hind legs of *C*. *Septempunctata* adults after the legs bilaterally amputated at 4th-instar larval stages. Phenotypes after half amputation (A) and complete amputation (C) are shown. In the two columns on the right, B and D are color-level inversion images of the leg segments highlighted, both partial regeneration (red mark) and complete regeneration (aqua mark) are shown. Each scale bar equals 500 μm.

After fore-, mid- or hind legs were bilaterally amputated, asymmetric phenotypes were observed. Two leg-regenerated phenotypes were detected in both half and complete amputations: 1) partial regeneration, leg was regenerated, but some segments were not regenerated, or were regenerated but were fused together (aqua marks of the leg segments highlighted), and significantly shortened segments were mainly detected at the distal tibia and tarsus; 2) complete regeneration, legs were regenerated with normal segments (red marks of the leg segments highlighted) (Fig 5). In a somite, the nutrition supplying the regeneration of bilateral legs via hemolymph was uneven [75], possibly leading to non-consistent sizes of regenerated legs.

### Potential investment tradeoff between leg regeneration and developmental cost

The leg regeneration triggered by cannibalism is common for coccinellid larvae in intensive rearing systems, but the mechanism of developmental delay and weight gain caused by leg regeneration remains unclear [76,77]. After leg amputation, the developmental cost of leg regeneration may decrease the competitive superiority and predatory agility of coccinellid larvae. What is worse, physical lesions may cause mutations during leg regeneration, disrupting cell multiplication and then triggering systematic delays [78,79]. Although developmental costs of leg regeneration cause negative effects, *C*. *septempunctata* larvae still choose to regenerate the lost leg completely to adapt to competitive environments. After larval cannibalism, leg-damaged ladybugs may tend to make an investment tradeoff between structural recovery and developmental cost, and this investment selection is essential to remaining competitive at the adult stage.

## Supporting information

S1 Table Data of figures used in this study. (XLSX)
